# Application of multivariate techniques for estimating herd feed efficiency using chemical and near-infrared calibration models in dairy cattle

**DOI:** 10.3168/jdsc.2025-0829

**Published:** 2025-09-17

**Authors:** Valentina Novara, Mattia Masseroni, Maddalena Canossa, Antonio Gallo

**Affiliations:** 1 Department of Animal Science, Food and Nutrition (DIANA), Università Cattolica Sacro Cuore, 29122 Piacenza, Italy; 2 A.I.A. - Agricola Italiana Alimentare S.p.A, 37142 Verona, Italy; 3 Romeo and Enrica Invernizzi Research Center for Sustainable Dairy Production of the Università Cattolica del Sacro Cuore (CREI), 29122 Piacenza, Italy

## Abstract

Feed efficiency (FE) is an indicator of overall farm nutritional efficiency, helping farmers to identify any critical points in nutritional management. Particularly, FE is a measure of the ability of animals to convert feed into milk and it can be influenced by genetic, health, management, and nutritional factors. Higher FE allows results in reduced feed and maintenance costs and contributes to improved economic and environmental efficiency of dairy farms. This study aimed to develop and compare 2 predictive models for estimating FE in dairy cattle using data derived from the TMR: one based on its chemical composition and the other on near-infrared (NIR) spectral data. A total of 144 TMR samples were collected from farms in Po Valley from 2021 to 2024 and analyzed with an Fourier-transform NIR spectrometer. The spectral data were processed with chemometric techniques, including least absolute shrinkage and selection operator regression, in order to build a predictive model of FE. The model based on chemical composition showed strong calibration performance (R^2^ = 0.80, SE of cross-validation [SECV] = 0.13) but decreased in external validation (R^2^ = 0.64, SE of prediction [SEP] = 0.11), indicating the presence of systematic bias. Conversely, the NIR-based model maintained more stable performance between calibration (R^2^ = 0.73, SECV = 0.16) and external validation (R^2^ = 0.70, SEP = 0.09), with lower slope distortion and offset. The results suggest that although chemical data offer high accuracy in controlled conditions, the NIR model may be more robust and generalizable for practical, on-farm prediction of FE, offering potential decision support. However, further improvements in calibration are needed to reduce systematic errors and increase the accuracy of the model.

## Highlights

• Predictive NIR spectroscopic models were developed to forecast FE using only TMR data.
• We confirmed NIR spectroscopy as a promising tool for rapid and cost-effective FE estimation.
• The models show promise but require further refinement.

## Graphical Abstract

**Figure:**
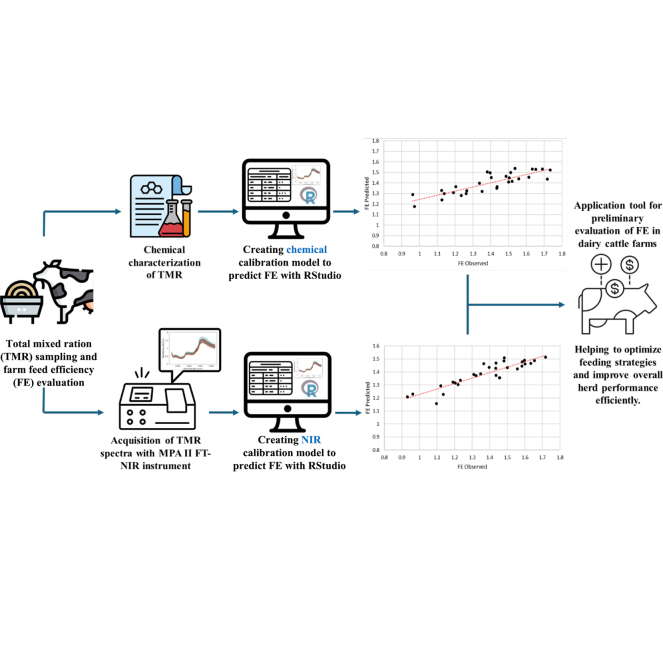
**Summary:** Near-infrared (NIR) spectroscopy is widely used in animal nutrition to evaluate the chemical composition of diets and feeds. In addition, its application is now also extensive and well proven for the evaluation of some indirect parameters, such as the digestibility of diets or animal efficiency. This study developed and compared 2 predictive models for estimating feed efficiency (FE) in dairy cattle using total mixed ration (TMR) data. The first model used the chemical composition of TMR, and the second applied NIR spectroscopy data analyzed with chemometric techniques. Both models showed good abilities to predict FE: The chemical model showed high accuracy in calibration but decreased performance in external validation, and the NIR model maintained stable predictive ability in validation. The greater robustness of the NIR model, as well as the fast and costeffective peculiarities of this approach, support its use for preliminary nutritional assessment in dairy systems. Icons made by authors from www.flaticon.com as follows: cow and table icons by Freepik; chemical analysis icon by imaginationlol; palette board icon by Prosymbols Premium; iMac icon by Those Icons; R icon by Becris; and cow icon by pbig.

Feed efficiency (**FE**) can be defined as the amount of milk produced relative to the amount of feed consumed (Bach et al., 2020), or in other words, it is a measure of the ability of dairy cows to convert feed into milk (Madilindi et al., 2022). Feed efficiency is considered a good predictor of the nutrient digestive efficiency (Puillet et al., 2016) and is essential for dairy farmers, being useful to identify inefficiencies in feeding practices and supporting decisions to improve herd performance and optimize resource utilization (Connor, 2015). According to Bellingeri et al. (2019), an average of 60% of the total expected costs of producing milk on a dairy farm can be attributed to animal diets, which include the costs associated with market or self-produced feed used for lactating cows, dry cows, heifers, and calves (St-Pierre and Glamocic, 2000). Increasing FE will lead to lower resource use or increased production, thereby diluting feed costs (Bach et al., 2020) and using less feed purchased from the market. This allows dairy farms to increase profits (Connor, 2015) by reducing feed costs while maintaining stable milk production (Madilindi et al., 2022). Nutritionists, through the use of software that is based on different animal nutritional models (**ANM**), must be able to formulate balanced diets that consider feedstuff prices and milk production prices (St-Pierre and Glamocic, 2000) so that the farm has an opportunity to remain competitive. In this context, improving FE in dairy cattle has become a critical objective, as it can lead to enhanced productivity, reduced feed costs, and a more sustainable and environmentally friendly milk production system. Consequently, optimization of efficiency in dairy systems results in decreased maintenance costs, as well as mitigation of environmental impacts, both at the farm level and globally (Pulina et al., 2020). By monitoring FE, nutritionists and farmers can make a preliminary but useful assessment of feed utilization in dairy cows based on production. The consequence is a range of benefits that cascade down to production, animal welfare, profits, and sustainability (Connor, 2015; Bach et al., 2020).

Feed efficiency is described as units of milk output per unit of feed input (Madilindi et al., 2022). On dairy farms, FE is generally evaluated at the group level for the whole herd, as is commonly done in field studies and benchmarking analyses (Atzori et al., 2021), where data on milk yield (**MY**) and DMI are more accessible in commercial dairy farms compared with individual measurements (Madilindi et al., 2022). However, even group-level intake estimation can be time-consuming. For this reason, researchers have explored the use of alternative methods, including mid-infrared (**MIR**) and near-infrared (**NIR**) spectroscopy (Lahart et al., 2019). Other studies have proposed prediction models based on MY and milk composition for estimating FE, as shown by Beard (2018), to simplify FE estimation under commercial conditions. Following similar thinking, this study aims to develop models for predicting the FE value of the herd based on another easily sampled and accessible matrix that influences it, such as nutritional composition and the NIR spectra of the TMR fed to dairy cattle located in different productive areas of Italy.

We hypothesized that because DMI, like MY, is part of the FE equation and is influenced by the chemical composition of the diet, FE could be predicted from the chemical characteristics of the TMR. Based on this and given our assumption that TMR chemistry may be related to FE, we further hypothesized that NIR spectroscopy, through its ability to estimate chemical composition quickly at low cost and with good repeatability or reproducibility of the results (Ghilardelli et al., 2022), could serve as an indirect tool for predicting FE. Therefore, we investigated whether FE could also be predicted directly from NIR spectral data without relying on intermediate chemical information. This approach can provide a practical method to assess herd efficiency without relying on data that are difficult to obtain on-farm.

To the best of our knowledge, there is a lack of studies in the literature referring to the estimation of FE based on diet alone.

A total of 144 TMR samples were collected from dairy farms in the Po Valley of northern Italy from 2021 to 2024, and their main chemical characteristics are reported in Table 1. Each sample was collected directly as described by Atzori et al. (2021). The TMR reflected typical diets used in intensive dairy farm systems within the sampling area and included forages such as corn silage, small grain silage (e.g., wheat and barley silage), haylage, alfalfa hay, grass hay, and concentrates. The TMR samples were brought to the laboratory within 12 h of sampling and were dried immediately in a 65°C forced-air oven (Consorzio Distribuzione Laboratorio, Italy) to a constant weight and then ground to a particle size of 1 mm in a rotor speed laboratory mill (Pulverisette 19, Fritsch, Germany). Chemical analysis of the dried TMR samples was conducted as previously described by Gallo et al. (2013). In addition to TMR sampling and analysis, data on the daily MY of the herd, corrected for 4% fat content (FCM, expressed in kg/d per cow), were extracted from the farm management system to calculate FE with the following formula (Atzori et al., 2021):

FE = FCM (kg/d per cow)/DMI (kg/d per cow).

The FE was calculated at the herd level as the ratio between the average 4% FCM yield per cow and the average DMI per cow, on a daily basis, using a practical, one-day assessment. The DMI was calculated as described by Gallo et al. (2022), corrected for DM content (Atzori et al., 2021), divided by the number of cows in the herd. The herds involved in the study showed the number of lactating cows ranged from 45 to 653 (median = 220), with DIM between 138 and 246 (median = 176). The proportion of primiparous cows ranged from 31% to 47% (median = 35%), an average of pregnancy rate (**PR**) of 23% (ranging from 17% to 41%), and an average calving-to-conception interval of 125 d (ranging from 86 d to 183 d).

**Table 1:** Main chemical characteristics and farm efficiency values in calibration and external validation datasets^1^

Item	Calibration dataset						External validation dataset					
	Mean	Maximum	Minimum	SD	Q1	Q3	Mean	Maximum	Minimum	SD	Q1	Q3
Chemical parameter												
CP, % DM	14.79	16.84	12.36	0.87	14.33	15.39	14.55	16.21	12.38	0.88	13.92	14.99
NDF, % DM	35.38	44.95	29.02	3.30	32.93	36.93	35.56	42.99	31.42	3.56	32.42	37.74
ADF, % DM	23.81	31.23	18.64	2.81	22.08	25.07	24.06	29.96	19.28	3.00	21.83	25.51
ADL, % DM	3.74	6.08	2.11	0.78	3.26	4.18	3.82	5.59	2.18	0.79	3.36	4.31
EE, % DM	3.25	4.29	2.04	0.54	2.83	3.64	3.34	4.01	2.20	0.62	2.83	3.92
Starch, % DM	25.65	31.34	16.87	2.93	24.11	27.54	25.47	30.05	19.84	3.03	23.63	27.78
Ash, % DM	7.77	9.85	6.45	0.67	4.15	5.22	7.65	9.31	6.45	0.76	7.25	8.03
Efficiency parameter												
MY, kg/d per cow	36.20	47.00	16.88	4.78	34.25	39.07	35.51	41.49	26.24	3.88	33.62	38.67
FCM, kg/d per cow	38.72	49.59	16.96	5.39	35.90	42.69	37.97	44.12	28.54	4.63	34.09	42.21
DMI, kg/d per cow	25.49	28.71	21.47	1.55	24.48	26.36	25.46	28.16	23.43	1.21	24.69	25.97
FE, dimensionless	1.41	1.79	0.93	0.16	1.29	1.51	1.38	1.71	1.14	0.15	1.25	1.46

1 EE = ether extract; MY = milk yield; FCM = milk yield corrected for 4% fat content; FE = feed efficiency; Q1 = 25th percentile; Q3 = 75th percentile.

Pearson correlation was calculated between the FE values (corrected for daily fat production) and the chemical parameters of the analyzed TMR to assess their degree of linear association. This preliminary analysis was useful in identifying which nutritional components could have a potential influence on FE and thus justify their inclusion in subsequent models.

An MPA II Multi-Purpose FT-NIR Analyzer (Bruker, Germany), which processes spectral data through Fourier-transform technique, was used to acquire the spectrum for each TMR sample. The sphere macrosample rotating cell was used to analyze the TMR sample. The read resolution was 8 cm^−1^, with a background scan number of 64, scanner temperature of 40°C, and sample temperature of 33.5°C. The obtained NIR spectra of each sample were reported in an Excel (2025, version 2502, Microsoft Corp.) matrix with the wavelength values and corresponding absorbance value, expressed as log (1/R), where R is the reflectance value. The databases were analyzed through univariate and multivariate approaches with RStudio (version 4.3.1, Posit Software).

The total of 144 TMR samples was divided into 2 groups: 120 samples were used for model calibration and internal validation, randomly split into a 75:25 ratio, with 90 samples for calibration and 30 samples for validation. The remaining 22 samples, collected later and excluded from model development, formed an external set to test predictive ability on independent data. For these samples, chemical composition and NIR spectra were obtained using the same procedures as for the development set.

Two separate databases were made, one consisting of the spectral data from TMR, and the other with chemical data. Principal component analysis was used on both databases to detect outliers according to the Mahalanobis distance (Parra-Forero et al., 2023) with a 1% limit. According to this technique, no sample is to be considered an outlier. The database formed by the spectral data was subjected to different preprocessing methods (Rinnan et al., 2009). Scattering was reduced through the application of the standard normal variate method, and spectral smoothing was performed through the application of Savitzky–Golay polynomial derivation with second-degree polynomial and window of 11 points.

Two prediction models were developed: one using TMR chemical composition and the other using NIR spectral data. A least absolute shrinkage and selection operator regression (Tibshiranit, 1996) was applied to the preprocessed dataset using the glmnet package in R Studio (Hastie and Stanford, 2016) by applying cross-validation with 10 folds.

Least absolute shrinkage and selection operator is a penalized linear regression method that imposes a constraint on the sum of the absolute values of the coefficients (Tibshiranit, 1996). By penalizing coefficients below a shrinkage parameter λ, it reduces some to zero, allowing feature selection of relevant variables as an efficient strategy to reduce the risk of overfitting (Ranstam and Cook, 2018) in NIR spectra. In this study, the optimal λ value was chosen as the one that minimized the generalization error during cross-validation. The general form of the prediction model was as follows:

*y_i_* = *β*_0_ + *β*_1_*X_i_*_1_ + *β*_2_*X_i_*_2_ + . . . + *β_n_X_ip_* + *ε_i_*,

where *y_i_* is the predicted FE, *X_i_*_1_ to *X_ip_* represent the selected chemical or NIR features associated with no-zero coefficients for observation *i*, *β_0_* to *β_p_* are the corresponding coefficients estimated after penalizing, and *ε_i_* represents the random error term.

The best final model from the tested ones was chosen (Aptula et al., 2005) according to the best combination between the calculated values of the coefficient of determination, the root mean square error, and mean absolute percentage error.

As the first step, the mean, SD, first and third quartile, and ranges for chemical and farm efficiency parameters were calculated for both the calibration dataset and the external validation dataset (Table 1). The reported data appeared typical both as average values and their distribution for TMR sampled in this specific production area (Atzori et al., 2021). Thereafter, a Pearson correlation matrix was constructed between FE and the main chemical characteristics of the diet. The results showed correlations of varying magnitude, suggesting the presence of linear relationships between some nutritional components and FE. In particular, moderate and significant (*P* < 0.05) positive correlations were observed for starch (r = 0.627) and fat (r = 0.526). Protein showed a weak positive correlation (r = 0.160). In contrast, the fiber fractions NDF (r = −0.684), ADF (r = −0.646), and ADL (r = −0.597) showed negative correlations with FE (*P* < 0.05). A negative correlation was also found for ash (r = −0.451). To further explore potential non-nutritional influences, Pearson correlations were also calculated between FE and same herd-level parameters collected by interviewing the farmers during the farm visit. The PR showed a positive correlation with FE (r = 0.519, *P* < 0.05), whereas the calving-to-conception interval was negatively correlated (r = −0.211, *P* < 0.05). Other parameters, including DIM, proportion of primiparous cows, number of lactating cows, and the proportion of pregnant cows, were not significantly associated with FE, likely because FE is a complex trait (Connor, 2015) influenced by multiple variables (Bach et al., 2020), so its association with herd data may be masked.

For both calibration models, the R^2^ value obtained in cross-validation was satisfactory (Table 2), indicating a good ability to explain variability in the data. The prediction model as a function of NIR data maintained its predictive ability even during the application of the model on the external database (R^2^ = 0.73 in cross-validation comparison with R^2^ = 0.70 in external validation), demonstrating discrete ability to adapt on unknown data. However, the model applied for FE prediction as a function of chemical parameters lost more in reliability on new data, with a reduction in R^2^ of more than 0.10 despite good model performance in cross-validation (R^2^ = 0.80). In the regression analysis between observed and predicted values on cross-validation, both models showed slope values greater than unity and marked negative offsets, indicating a tendency to overestimate the variation in the data and the presence of systematic bias. However, in external validation, the NIR model showed better generalization ability, with slope of 1.19 and offset of −0.30, significantly closer to ideal, suggesting greater prediction reliability on unknown data than the chemical model, which maintained a high slope (value = 1.75) and negative offset (value = −1.10). However, the relationship between observed and predicted values remained quite well defined, with a good ability to distinguish between high and low values.

**Table 2:** Chemical and near-infrared spectroscopy parameters from cross-validation and prediction^1^

Item	Cross-validation					External validation				
	R^2^	RMSE	SECV	Slope	Offset	R^2^	RMSE	SEP	Slope	Offset
Chemical	0.80	0.12	0.13	1.91	−1.27	0.64	0.11	0.11	1.75	−1.10
NIR	0.73	0.16	0.16	2.05	−1.48	0.70	0.09	0.09	1.19	−0.30

1 RMSE = root mean square error; SECV = SE of cross-validation; SEP = SE of prediction.

The intricate relationship between TMR composition and FE in dairy cattle represents a cornerstone of modern dairy production, directly influencing profitability and sustainability. For this reason, this study examined the prediction of FE from the chemical composition and NIR spectra of TMR. We started by investigating the correlations between FE and TMR chemical parameters to identify the variables that most strongly influence FE and to assess if a predictive model could be applied. Chemical components influence dynamic intake, fermentation patterns, and metabolic efficiency, and consequently how animals convert feed into milk (Bach et al., 2020). Moreover, as noted by de Ondarza and Tricarico (2017), herd structure variables can influence FE prediction. However, by sampling across multiple farms, we aimed to capture a wide range of variability (Lahart et al., 2019), which is crucial to ensure applicability under field conditions, as highlighted by McParland and Berry (2016).

Beard (2018) attempted to estimate intake conversion ratio from milk data, considering daily production and milk composition. The prediction equations showed an R^2^ value of 0.44. We explored a complementary approach by developing a predictive model for FE based on the chemical composition of the TMR. Because TMR composition directly contributes to the calculation of FE, it represents a potential predictor of this trait, and the model demonstrated good predictive performance, although with some systematic errors. In the study of Shetty et al. (2017), the predictive ability of DMI models based on MY and milk composition improved slightly when MIR spectral data were included, with R^2^ values increasing to 0.81. These results suggest that MIR spectra may add some information to prediction models, but also raise the question of whether this information goes substantially beyond that offered by milk composition alone in predictive models. Building on this, and considering that DMI is a trait of FE, we further assumed that the NIR spectrum of TMR, being directly related to the ingested feed, could carry sufficient information to independently predict FE, although the use of NIR spectra to predict directly FE is still underexplored because the technique is relatively recent (Madilindi et al., 2022). Supporting this hypothesis, Lahart et al., (2019) demonstrated the potential of MIR milk spectra to predict DMI. Likewise, Parra-Forero et al., (2023) obtained strong predictive performance using NIR spectra from fecal samples and obtained comparable R^2^ values both in calibration (R^2^ = 0.94) and cross-validation (R^2^ = 0.82). Furthermore, in this study also performed external validation with 22 samples not used in the reference library (R^2^ = 0.86). This reinforces the idea that NIR spectral data from different biological matrices may contain useful information for predicting intake-related traits. Therefore, it is reasonable to assume that the NIR spectrum of the TMR could also carry useful information for estimating DMI, suggesting that spectral data, when linked to appropriate biological variables, may allow for indirect estimation of FE. As emphasized by Tedeschi (2019), ANM proved to be a reliable and robust tool for formulating nutritionally balanced rations, effectively matching dietary inputs to animal requirements across a wide range of production contexts (INRA, 2018; NASEM, 2021; Cornell Net Carbohydrate and Protein System, Van Amburgh et al., 2015). Nutritionists apply these models to formulate chemically balanced rations. Therefore, using NIR spectroscopy for the chemical assessment of the TMR appears to be an effective approach for rapidly and reliably estimating FE. To date, existing studies try to estimate FE traits using different biological matrices or residual feed intake (**RFI**) starting from Fourier-transform infrared spectra of milk samples. This concept was highlighted by McParland et al. (2014) who used RFI as a measure of FE in dairy cattle and attempted to predict it using partial least squares regression applied to MIR spectra of morning and evening milk samples, obtaining R^2^ values comparable to those reported in the present study. Specifically, for morning samples, they reported R^2^ = 0.50 in cross-validation and 0.48 in external validation with slope = 0.93, whereas for evening samples, they achieved R^2^ = 0.60 in cross-validation and R^2^ = 0.58 in external validation, with slope = 0.88.

The data in the present study suggest that although the model based on chemical analysis appears to perform better at calibration, the model based on NIR spectroscopy is more suitable for predictive use on external data, due to better consistency between the observed and predicted values and lower systematic bias. The ability of both models to properly distinguish between high and low FE values represents a baseline for further improvements.

As highlighted by de Ondarza and Tricarico (2017), several on-farm FE indices, including the one used in this study, have inherent limitations and must be interpreted within the specific context of each dairy system. Nevertheless, the approach adopted here remains a practical and informative indicator for on-farm applications. In line with this perspective, the present study was based on data collected from a wide range of commercial dairy herds, intentionally capturing variability related to nutrition, management, genetics, and environmental traits characterizing dairy farm enterprises. The approach proposed in the present study differed from most studies, which were typically conducted on individual animals under controlled experimental conditions, where such variability is often intentionally minimized (McParland and Berry, 2016). Our approach, instead, aimed to reflect the complexity of real intensive dairy farming systems with all the variability that can be found.

The present study should be considered a preliminary evaluation of the use of NIR spectroscopy to predict FE. The performance of the model could be further improved by adding more data, milk spectra, and increasing the sample size by including TMR from a more extensive geographical area, as well as including other economic and nutritive indicators, as prediction models based only on MIR spectra have shown a tendency for lower accuracy compared with those integrating additional variables (Madilindi et al., 2022). Additionally, testing alternative predictive models and exploring different spectral preprocessing methods may improve accuracy and improve the characterization of this type of data, and incorporating herd composition parameters may influence the robustness of prediction models.

In conclusion, the 2 different models presented in this study demonstrated good predictive ability for FE using TMR samples. These models could provide useful information for quickly and accurately assessing animal FE, helping to optimize feeding strategies and management practices while improving overall herd performance. Additionally, NIR spectroscopy offers the significant advantage of being fast and inexpensive. However, further improvements are necessary, particularly to reduce the current systemic error.
